# Mediating Effect of Trait Emotional Intelligence Between the Behavioral Activation System (BAS)/Behavioral Inhibition System (BIS) and Positive and Negative Affect

**DOI:** 10.3389/fpsyg.2019.00424

**Published:** 2019-03-05

**Authors:** Ana Merchán-Clavellino, Jose Ramón Alameda-Bailén, Antonio Zayas García, Rocio Guil

**Affiliations:** ^1^Psychology Department, University of Cádiz, Cádiz, Spain; ^2^INDESS (University Institute for Sustainable Social Development), University of Cádiz, Cádiz, Spain; ^3^Department of Clinical and Experimental Psychology, University of Huelva, Huelva, Spain

**Keywords:** emotional intelligence, TMMS-24, positive affect (PA), negative affect, reinforcement sensitivity theory, BIS/BAS

## Abstract

Gray (1970, 1981, 1987) proposed a behavioral motivation theory (Reinforcement Sensitivity Theory, RST), which describes the Behavioral Activation/Approach System (BAS) and the Behavioral Inhibition System (BIS). Some studies relate higher activation of BAS to positive affect, whereas BIS activation is linked to negative affect, particularly to high levels of anxiety and depression. Research data suggests that greater Trait Emotional Intelligence (TEI) influences optimal development of well-being and psychological adjustment, such as positive affective states. However, a recent study relates the motivational BIS/BAS systems with TEI, showing that high TEI is characterized by sensitivity to reward (BAS), and low TEI due to activation of the BIS system. The aim of this study was to explore how TEI may mediate the relationship between BIS/BAS sensitivity and positive and negative affect. Four-hundred and sixty-seven undergraduate students (385 females) were evaluated. TEI was evaluated with the Trait Meta-Mood Scale (TMMS). Affective states were measured with the Positive (PA) and Negative Affect (NA) Schedule, and BIS/BAS sensitivity was measured with The Sensitivity to Punishment (SP) and Sensitivity to Reward (SR) Questionnaire. The results reveal the influence of the two motivational systems on affective states, and show how this relationship is modified by and better explained through TEI. That is, a stronger approach to appetitive stimuli produces more positive affect, but a belief that one [does not] understand unpleasant emotions or that one analyzes them, or thinks that one cannot regulate or control emotions will reduce that positive state. Greater activation of inhibitory behaviors will produce greater negative affect, and this will increase when one perceives that one attends excessively to one's feelings or does not understand them or feels incapable of regulating them. Accordingly, although motivators could be a focus of interest for intervention, this study shows that the efficiency and profitability of these practical applications increases by adding TEI.

## Introduction

From a neurobehavioral perspective, individual differences in personality traits emerge from the activity of certain brain systems. Eysenck's model (1967), perhaps the most representative within this perspective, is based precisely on the identification of a series of personality traits that are independent of each other: Extroversion-Introversion and Neuroticism-Stability. Different neural structures and mechanisms are related to the psychological differences associated with these traits. Thus, extroversion-introversion would be determined by the reactivity of the central nervous system; and neuroticism-emotional stability would be controlled by the cortical-limbic loop that connects the cerebral cortex with the autonomous nervous system (Eysenck, 1967).

The Reinforcement Sensitivity Theory (RST; Gray, 1970, 1981, 1987) incorporates motivational aspects in the explanation of personality, remodeling Eysenck's theoretical proposal and drawing on the fact that emotional situations are not only characterized by the intensity of the emotional arousal, but also by the motivational direction of the behavior depending on the appetitive or aversive signals present. This theory constituted a strong impulse for the biological study of personality, associating individual differences in diverse personality traits with variations in the reactivity of neurobehavioral systems related to motivational, emotional, and learning processes (Depue and Collins, 1999).

RST implies the existence of different neural systems specialized in detecting, processing, and responding to certain stimuli. Each type of stimulus will launch a specific neural system, activating motivational and emotional states, behavioral responses, etc (Corr, 2008a). Each specific brain system would be responsible for controlling concrete behaviors and emotions that would be associated with certain perceptions or cognitions (Corr, 2008b), while emphasizing emotional intensity and motivational direction. In turn, these systems could be interconnected to more general functions in broader contexts and modulated by general systems (like those of arousal and attention). Thus, for Gray, personality would be the outcome of different neural systems reactivity, and this could explain individual differences (Gray, 1970, 1982, 1993).

RST postulates two key dimensions related to Eysenck's model: Anxiety, ranging from the pole of Extroversion-Stability (low anxiety) to Introversion-Neuroticism (high anxiety); and Impulsivity, which ranges from the Introversion-Stability pole (low impulsivity) to Extroversion-Neuroticism (high impulsivity). The level of impulsivity is directly related to sensitivity to cues of reward and absence of punishment, whereas levels of anxiety are related to sensitivity to cues of punishment, no-reward, and novelty. Gray proposes that behaviors are dually determined by their sensitivity to cues related to the onset of positive reinforcement and their sensitivity to cues related to punishment. These sensitivities are governed, respectively, by two different brain systems, explaining responses in the face of positive or negative stimuli.

### Behavioral Inhibition System/Behavioral Arousal System

On the one hand, the Behavioral Arousal System (BAS) “Let's go for it,” is the brain system responsible for responding to conditioned and unconditioned stimuli that cue reward (appetitive) or the absence of punishment. When one of these stimuli is present, two effects occur in BAS-mediated behavior: a motivational effect, due to an increase in the arousal, which stimulates and redirects behavior along a spatial-temporal gradient toward the source of reinforcement; and an effect of learning, which redirects attention toward the reward stimulus, facilitating information processing and learning stimulus-stimulus and stimulus-response relations (Pickering and Gray, 2001; Pickering and Smillie, 2008). BAS helps to identify cues associated with positive reinforcement (and absence of punishment) and allows assigning value to the reinforcing stimuli present. BAS activity depends on the dopaminergic system and is composed of two interrelated subsystems: the dorsal striatum (caudate and putamen) and the ventral striatum (core accumbens). BAS activity is related to the development of positive affect or mood and impulsivity (Gray, 1987; Corr, 2004). BAS uses a series of processes (different, but related) to achieve its goals, such as reward reactivity and impulsivity as it approaches and captures the final reinforcer (Corr and Cooper, 2016). BAS arousal leads to the experience of hopeful excitement, it drives persistence to achieve the desired goals and a sense of joy when they are attained.

On the other hand is the Behavioral Inhibition System (BIS), which helps the organism to identify cues associated with punishment or the onset or negative events, assigns value to aversive events (other authors have also proposed similar ideas, for example Konorski, 1967; Lang et al., 1992). Like BAS, it is a feedback device (in this case negative), reacting to conditioned aversive stimuli and responding to cues of punishment, no-reward, or new stimuli. It acts by suppressing behavioral performance, and increases attention to the environment and novelty, so that the next action (identical or not to the interrupted action) is performed with greater intensity and speed. At the cognitive level, BIS predicts the next most likely event and compares it with the current event. The brain structures related to BIS are the septo hippocampal system, its monoaminergic afferents, and its neocortical projections toward the prefrontal cortex. BIS activity has been associated with the development of negative affect or mood and anxiety (Gray, 1987; Corr, 2004). BIS arousal is related to behaviors of passive avoidance, contributing to: the evaluation of risk and rumination, which may lead to the experience of anxiety. In summary, whereas BAS has been associated with the experience of positive affect (PA), BIS is related to the experience of negative affect (NA) (Corr, 2008a,b).

Various self-report instruments have been developed to study the individual differences in BIS/BAS reactivity. Initially, some authors have used Eysenck's Personality Inventory (Eysenck and Eysenck, 1975), assuming that the scales of Extroversion and Impulsivity of that instrument are equivalent to the BAS construct, and that the scale of Neuroticism measures something “rather similar” to BIS. Other researchers, in contrast, have sought to develop their own instruments to assess the BIS/BAS profile, such as the Appetitive Motivation Scale (Jackson and Smillie, 2004) or the Generalized Reward and Punishment Expectancy Scales (Ball and Zuckerman, 1990; Corr, 2001). But the most widely used are the Behavioral Inhibition/Behavioral Activation System Scales (BIS/BAS Scales; Carver and White, 1994) and the Sensitivity to Punishment and Sensitivity to Reward Questionnaire (SPSRQ; Torrubia et al., 2001).

Originally, BIS-BAS are functionally independent, although in one development of the theory, Gray and McNaughton (2000) proposed their interdependence, turning the mechanism of avoidance into a Fight-Flight-Freeze System (FFFS), which modulates reactions to all aversive stimuli (conditioned or not) and produces avoidance and escape behaviors; high FFFS reactivity is associated with levels of fear and avoidance behaviors. According to Gray and McNaughton, BAS remains relatively unchanged, mediating the reactions to appetitive stimuli and approach behavior, whereas BIS is responsible for detecting and resolving conflict (between approach and avoidance), beyond sensitivity to punishment itself.

Individual differences in personality and behavior would be based on differences in the reactivity of these systems, such that an individual may be highly sensitive in both systems, whereas others may have greater sensitivity in one of the two systems. Different types of BIS/BAS sensitivity is associated with specific clinical pathologies. For example, people with a particularly sensitive BIS are prone to present problems of anxiety and depression (Johnson et al., 2003; Leen-Feldner et al., 2004; Maack et al., 2012; Hundt et al., 2013), a tendency to worry or to anxious rumination (Corr and McNaughton, 2008) as the result of excessive attention to cues related to negative events. High BAS reactivity is associated with orientation toward reward and impulsivity. BAS is also linked to positive affect (Meyer and Hofmann, 2005). Hence, people with a very sensitive BAS and not very skilled at identifying cues associated with punishment would be particularly vulnerable to the development of addictive behaviors (Knyazev, 2004; Pardo et al., 2007; Hundt et al., 2008) as a result of excessively valuing the immediate reinforcing properties, but not adequately appraising the long-term effects.

### Affective States and Emotional Intelligence

Affective states are conceptualized as two independent dimensions or factors that determine emotional experiences. On the one hand, positive affect (PA) indicates that an individual feels excited, alert, and active and, on another hand, negative affect (NA) may reflect fatigue, sadness, and mental and physical exhaustion (Watson et al., 1988; Sandín et al., 1999; Gray and Watson, 2007). The expression of this type of experiences is considered important in physical, emotional, and social health risk prevention. Certain disorders, such as anxiety and depression, share high levels of negative affect, whereas low levels of positive affect are only related to depression (Sandín et al., 1999). For this reason, research must incorporate the study of both dimensions.

When referring to physical, emotional, and social health risk prevention, many studies consider emotional intelligence (EI) as a transcendental variable, as it has been determined that individuals with adequate perception of emotion management present optimal development of well-being and good psychological adjustment (Petrides et al., 2007; Schutte et al., 2007; Martins et al., 2010; Salguero et al., 2011; Fernández-Berrocal et al., 2012; Mestre et al., 2017) and of low levels of negative affect and high levels of positive affect (Gohm and Clore, 2002; Palmer et al., 2002; Extremera and Fernández-Berrocal, 2005; Extremera and Rey, 2016).

The term EI was coined by Salovey and Mayer (1990). These authors postulate the structure of EI as a model of four branches or interrelated skills, comprising the skill to perceive, appraise, and express emotions; the skill to access and/or generate feelings that facilitate thought; the skill to understand emotions; and the skill to regulate emotions (Mayer and Salovey, 1997).

Since this first approach, other models have emerged, attempting to conceptualize EI from different perspectives (Mestre and Fernández-Berrocal, 2007). They can be classified generally as skills models and mixed or trait models. Skills models consider EI as the ability to process emotional information to improve and guide thoughts. Mixed or trait models consider EI as stable personality traits, behavioral tendencies, and self-perceived abilities (Petrides, 2010).

Different methods to assess the EI construct have emerged over the years. The most commonly methods used are self-report questionnaires and evaluations by observers or 360° (Extremera et al., 2004; Mestre and Guil, 2006). Specifically, among the self-report instruments, the most widely used has been the Trait Meta-Mood Scale developed by Salovey et al. (1995). This meta-knowledge trait scale provides a perceived EI index or Trait Emotional Intelligence (TEI), reporting people's perception of their skills to attend to, clarify, and repair their own emotional states.

### BIS/BAS and EI

As RST is a neuropsychological theory that expresses the personality in terms of emotion, motivation, and learning (Corr, 2008a), it seems to be the most adequate framework to investigate EI. Bacon and Corr (2017) proposed the first study, empirically confirming the relations between EI and RST, reporting that people with low EI are more restless and take more precautions in rewarding environments, whereas people with high EI experience less motivational conflict (Corr, 2008a). Bacon and Corr also observed that people with high EI are more positive and more resilient, they are characterized by being goal driven (BAS) and they experience lower levels of negative feelings like fear, frustration, or sadness (BIS).

Within the EI dimensions, Self-control is the most closely related to the RST variables. People who obtain high scores are more likely to regulate their emotions and behaviors effectively, concentrate on achieving their goals, be more receptive to the perspective of rewards for their efforts, but they would not act impulsively to obtain them. Other EI dimensions, such as Well-being, Emotion, and Sociability, are also positive in terms of attitude, affect, and relations with others, contributing to the link established between EI and favorable life results, but they are less related to the RST (Bacon and Corr, 2017).

### BIS/BAS, EI and Affective States

In short, only the study of Bacon and Corr (2017) showed that high TEI is characterized by sensitivity to reward (BAS) and low TEI by BIS activation and previous studies (Meyer and Hofmann, 2005; Hundt et al., 2013; Li et al., 2015) observed BIS was associated with negative mood and emotions whereas BAS was associated with positive experiences. Due to these findings, two independent models have been planned, one for each system proposed by the RST. But we do not know how TEI mediates the relation between the motivational systems (BIS/BAS) and affective states.

Hence, the goal of this study is to explore how TEI (Attention, Clarity, and Emotional Repair) can mediate the relation between BIS/BAS and Positive and Negative Affect. For this purpose, two mediation models were designed: Model A examines the effect of SR or BAS on PA, and Model B examines the effect of SP or BIS on NA. Both mediation analyses were performed using TEI as the mediator. In the first model, we hypothesized that greater BAS activation would be associated with higher levels of EI, which, in turn, would be associated with greater PA. In the second model, our hypothesis was that greater BIS activation would be associated with lower levels of EI, which, in turn, would be associated with greater NA.

## Materials and Methods

### Participants

The sample consisted of 467 undergraduate students, 385 women (82.4%) and 82 men (17.56%). The mean age was 21.79 years (*SD* = 5.19). The participants were from the University of Huelva, Spain. They were studying Psychology (87.15%), Psychopedagogy (12.43%), Social Education (0.21%) and Tourism (0.21%). A total of the sample 57.6% were studying first, 8.99% second, 13.28% third, 16.06% fourth and finally 4.07% fifth.

### Procedure

Participation in the study was voluntary and confidential. The study was carried out in compliance with the Declaration of Helsinki, and all participants signed the informed consent. The students completed the different online self-report questionnaires and were rewarded with course credits.

### Measures

#### Trait Meta-Mood Scale (Salovey et al., 1995)

TEI was evaluated by the Spanish version of the Trait Meta-Mood Scale (TMMS; Fernández-Berrocal et al., 2004). This questionnaire evaluates the perception of or beliefs about one's emotional abilities. This scale contains 24 items, rated on a 5-point Likert scale ranging from 1 (*strongly disagree*) to 5 (*strongly agree*). It is divided into three dimensions: Emotional Attention (ability to identify one's own emotions and those of others and know how to express them), Emotional Clarity (understanding emotions), and Emotional Repair or Regulation (ability to handle emotions), with each dimension containing 8 items. The reported reliability and validity indexes are adequate (Fernández-Berrocal et al., 2004). In our sample, Cronbach's alpha for each dimension was as follows: Emotional Attention α = 0.88; Emotional Clarity α = 0.90, and Emotional Repair α = 0.87.

#### The Sensitivity to Punishment and Sensitivity to Reward (Carver and White, 1994)

BIS/BAS sensitivity was measured by the Spanish version of The Sensitivity to Punishment (SP) and Sensitivity to Reward (SR) Questionnaire (SPSRQ; Torrubia et al., 2001). It consists of 48 dichotomous items (Yes-No) and is divided into two scales: Sensitivity to Punishment (SP), which consists of 24 items considered measures of BIS, and Sensitivity to Reward (SR) as a measure of BAS. The reliability of the scale is adequate, with the SP scale showing an alpha of 0.83 and the SR scale an alpha of 0.76 (Caseras et al., 2003). In this sample, the reliability indices were 0.78 for the SP subscale and 0.77 for the SR scale.

#### Positive and Negative Affect Schedule (Watson et al., 1988)

Affective states were measured through the Spanish version of the Positive (PA) and Negative Affect (NA) Schedule (PANAS; Sandín et al., 1999). This scale is a widely used self-report measure, developed to evaluate these two dimensions independently. The original version presents adequate indicators of internal consistency for both dimensions (Watson et al., 1988). Each sub-factor showed adequate reliability in the present sample: PA α = 0.88 and NA α = 0.82.

### Statistical Analysis

The analyses were carried out using the SPSS package (version 20.0; IBM, Chicago, IL). In the preliminary analyses, descriptive statistics and internal consistency were calculated with Cronbach's alpha. Pearson correlations between the study variables were also calculated, and Student's *t*-test was used to determine sex differences. In order to verify the influence of sex and age on the proposed models, linear hierarchical regressions were performed in which sex and age were entered first (as control variables). All mediation analyses described below were estimated with the PROCESS macro (Hayes, 2013) using SPSS 20 software. We used Model 6 to examine the direct and indirect effect of two mediation models; Model A examines the effect of SR or BAS on PA, and Model B examines the effect of SP or BIS on NA. Mediation analyses were conducted using TEI as a mediator. To verify which of the indirect effects was the most important, we performed specific contrasts for indirect effects. As a statistical significance criterion, we used the 95% confidence interval (CI) generated by the bias-corrected bootstrap method set to 10,000 reiterations.

## Results

### Preliminary Analyses

Table 1 presents the descriptive statistics of the research variables and their internal consistency.

**Table 1:** Descriptive statistics and Cronbach's α values of sensitivity to punishment/sensitivity to reward, positive affect*/*negative affect, and trait emotional intelligence.

	***M***	***SD***	**Cronbach's α**
Emotional attention	29.14	5.21	0.88
Emotional clarity	29.05	5.4	0.90
Emotional repair	29.26	5.64	0.87
Sensitivity to punishment	10.78	5.13	0.78
Sensitivity to reward	9.75	4.25	0.77
Negative affect	19.03	7.24	0.88
Positive affect	29.57	6.6	0.82

Table 2 shows the Pearson correlations among the main variables in our study. According to Model A, Sensitivity to Reward was positively associated with Positive Affect and negatively with Emotional Clarity in all participants. Moreover, Positive Affect had positive associations with all TEI dimensions. For Model B, Sensitivity to Punishment showed significant positive relations with Negative Affect and Emotional Attention and negative associations with Clarity and Emotional Repair. Negative Affect was also negatively associated with Clarity and Emotional Repair and positively with Emotional Attention. Finally, results showed significant positive associations between Attention and Clarity and between Clarity and Emotional Repair.

**Table 2:** Pearson correlations among sensitivity to punishment/sensitivity to reward, positive affect/negative affect, trait emotional intelligence, and age.

	**1**	**2**	**3**	**4**	**5**	**6**	**7**
1. Emotional attention	–						
2. Emotional clarity	0.13^**^	–					
3. Emotional repair	0.01	0.31^**^	–				
4. Sensitivity to punishment	0.25^**^	−0.33^**^	−0.35^**^	–			
5.Sensitivity to reward	0.05	−0.13^**^	−0.02	0.06	–		
6. Negative affect	0.30^**^	−0.27^**^	−0.28^**^	0.30^**^	0.17^**^	–	
7. Positive affect	0.15^**^	0.18^**^	0.35^**^	−0.21^**^	0.18^**^	0.29^**^	–
8. Age	−0.04	0.13^**^	0.07	−0.16^**^	−0.09	0.06	0.07

** *p < 0.001; N = 467*.

Table 3 presents the descriptive statistics for men and women. There were statistically significant sex differences for the SR and PA variables.

**Table 3:** Descriptive statistics for men and women and Student's *t*-test.

	**Females**		**Males**		***t***	***gl***	***p***
	***M***	***SD***	***M***	***SD***			
Emotional attention	29.23	5.27	28.68	4.91	0.87	465	0.385
Emotional clarity	28.98	5.51	29.37	4.87	−0.581	465	0.562
Emotional repair	29.19	5.83	29.61	4.69	−0.704	139.88	0.483
Sensitivity to punishment	10.87	5.03	10.37	5.59	0.812	465	0.417
Sensitivity to reward	9.4	4.2	11.38	4.15	−3.88	465	0.000^**^
Negative affect	18.89	7.37	19.71	6.61	−0.93	465	0.353
Positive affect	29.26	6.71	31	5.87	−2.17	465	0.030*

** p < 0.001; *p < 0.05.

Due to the sex differences and the significant correlation between age and Emotional Clarity, various linear regression analyses were carried out to verify the influence of these variables in the two proposed models. In Model A, we determined whether SR and the TEI dimensions are related to PA after controlling for the influence of sex and age. The model generated was significant, *F*_(6, 460)_ = 17.83, *p* = 0.000, with an adjusted *R*^2^ = 0.189, but neither sex nor age were associated with PA (*p* > 0.05). For Model B, another regression analysis was performed to determine whether SP and the TEI dimensions are related to NA after controlling for the influence of age. Analyses showed a significant model, *F*_(5, 461)_ = 28.25, *p* = 0.000, with an adjusted *R*^2^ = 0.235. Results show that age was not associated with NA (*p* > 0.05).

### Mediation Analyses

In this study, for Model A, SR was considered the first variable (predictor, *X*) and PA as the outcome (*Y*). In Model B, SP was considered the first variable (predictor, *X*) and NA the outcome (*Y*). Emotional Attention (*M*_1_), Emotional Clarity (*M*_2_), and Emotional Repair (*M*_3_) were considered the mediator variables for both models.

**Figure 1:**
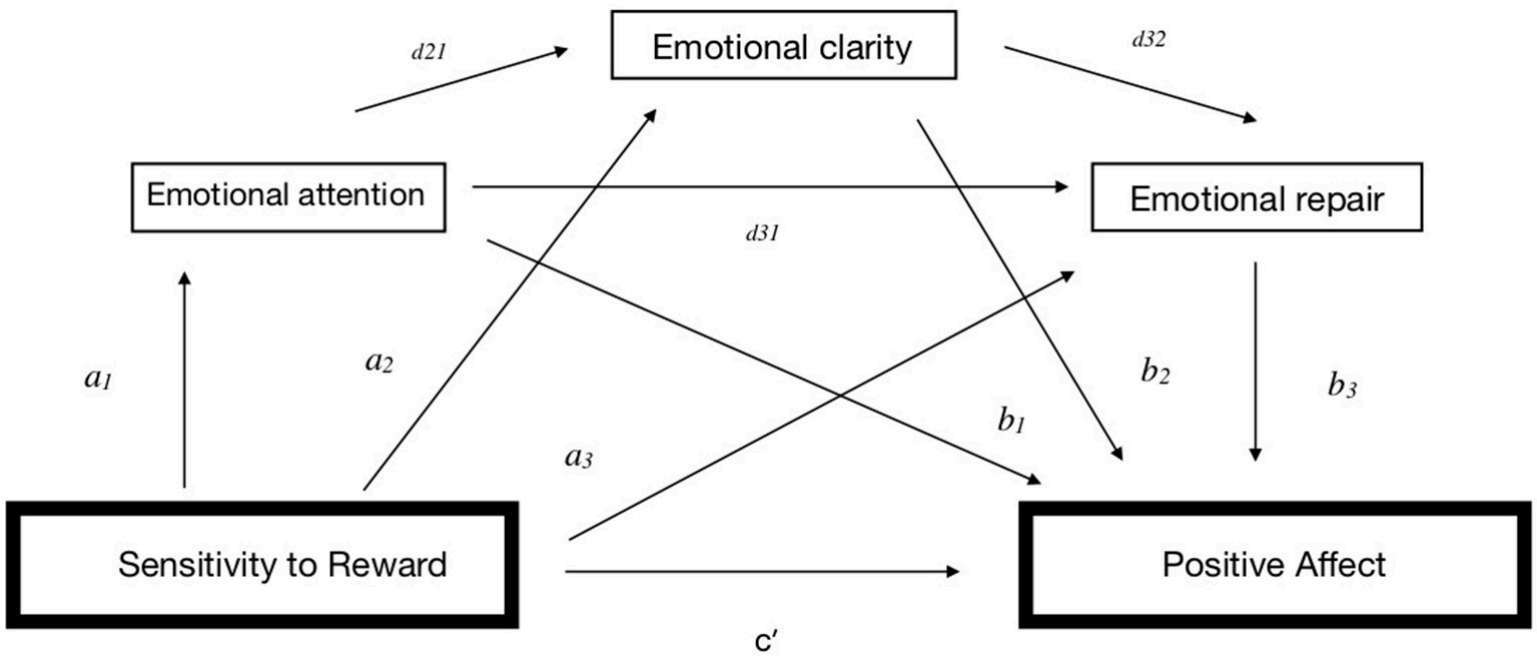
Indirect effects for Model A.

**Figure 2:**
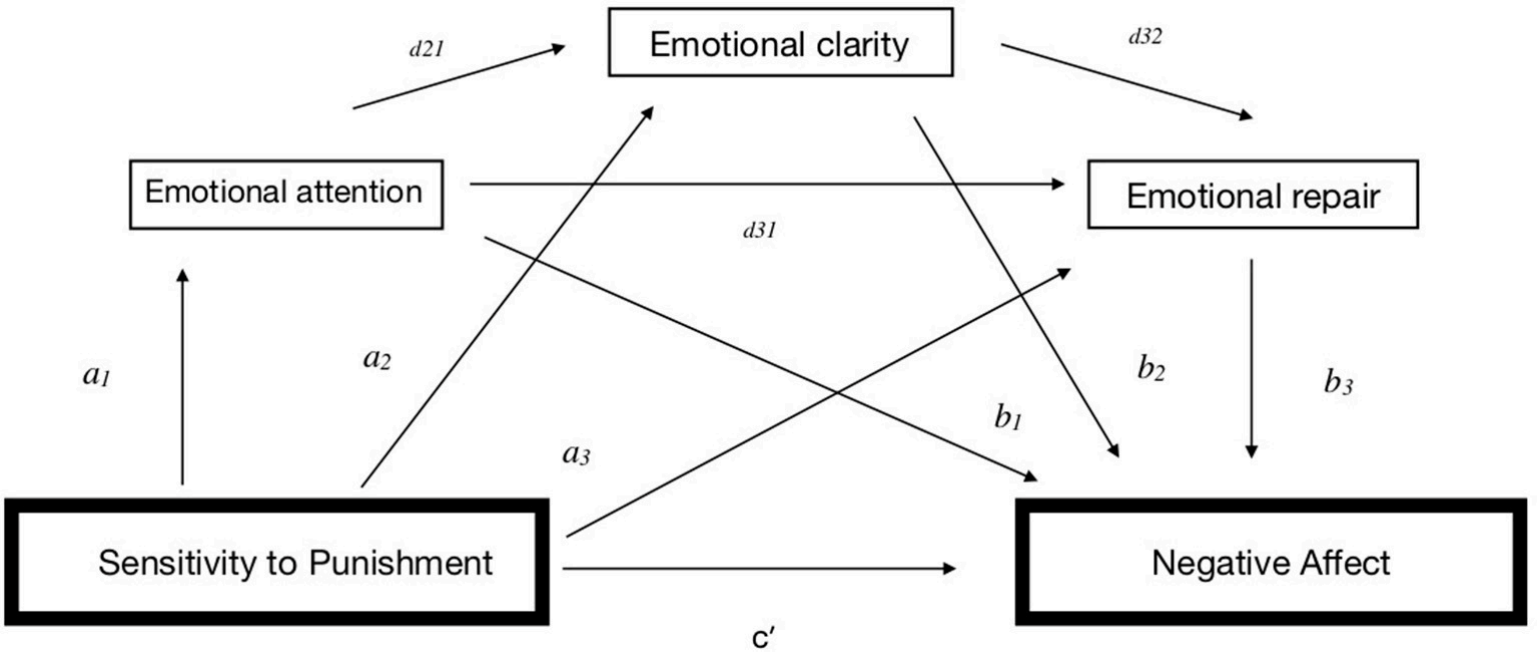
Indirect effects for Model B.

As illustrated in Figures 1, 2, total effect (*c*) refers to the relationship between SR/SP and PA/NA, respectively, without controlling for the mediators; direct effect (*c*′) refers to the relationship between SR/SP and PA/NA, respectively, after controlling for the mediators; total indirect effect (*a*) represents the association between the predictors SR/SP and three mediators (*a*_1_*, a*_2_, and *a*_3_); and total indirect effect (*b*) refers to the role of the three mediators in the relationship with PA/NA, respectively (*b*_1_*, b*_2_, and *b*_3_). Total indirect effect (*d*) refers to the relationship of the three mediators with each other (*d*_21_*, d*_32_, and *d*_31_), and specific indirect effect (*a*_1_*b*_1_, *a*_2_*b*_2_, and/or *a*_3_*b*_3_) refers to the role of a specific mediator in the relationship between SR/SP and PA/NA, respectively.

The first model (A) evaluated the possible mediation of TEI (Attention, Clarity, and Repair) in the relationship between SR and PA. In the first regression, SR accounted for 3.09% of the unique variance in PA (*R*^2^ = 0.03098, *F* = 14,824, *p* < 0.01). However, 18.17% of the total amount of variance was accounted for by the global model, which included SR and the three proposed TEI mediators (*R*^2^ = 0.1817, *F* = 35,956, *p* < 0.01).

**Figure 3:**
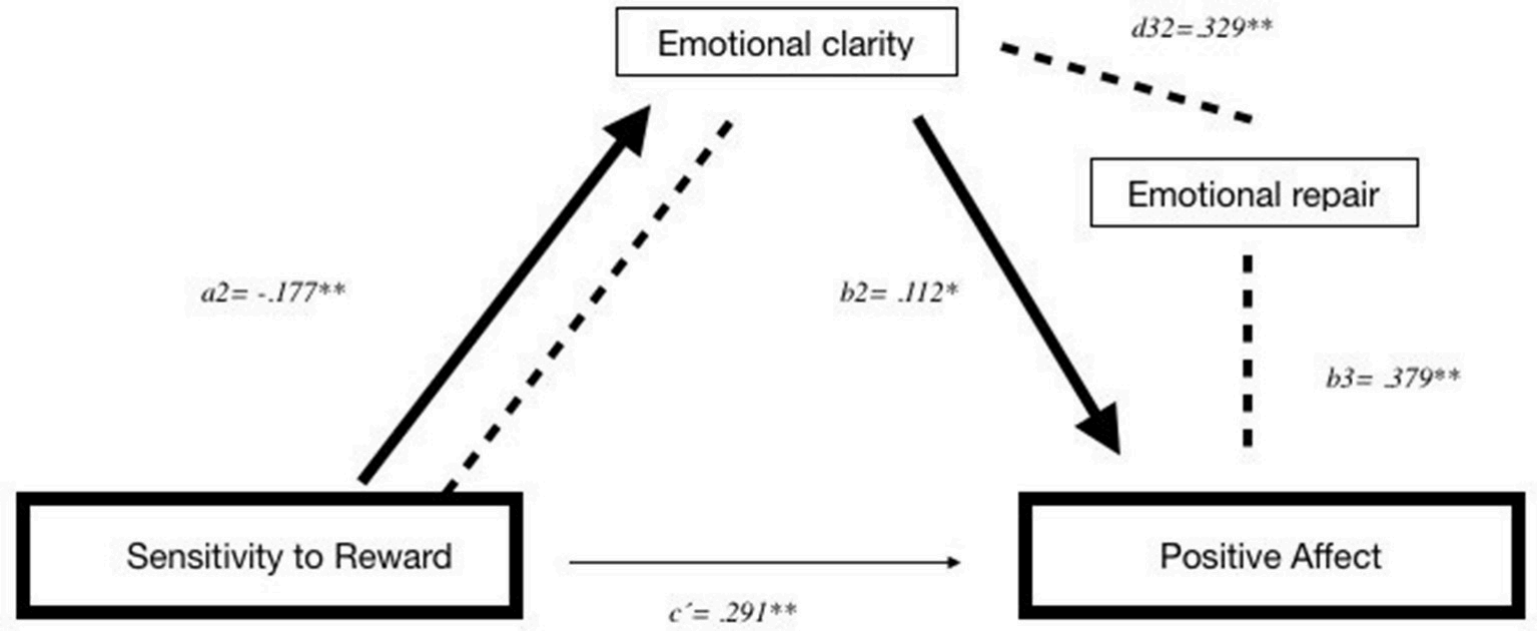
Illustration of two-way of serial mediation model between sensitivity to reward and positive affect. **p < 0.001; *p < 0.05.

The values provided in Table 4 show that the total effect (*c*) and the direct effect (*c*′) of SR on PA were significant. As the regression coefficient estimates, based on the 95% CI of the point estimate did not contain zero—evidence of the mediation of indirect effects—, we obtained two specific indirect effects through (1) the Emotional Clarity relationships (Ind5 = *a*_2_*b*_2_), where greater SR was associated with lower Emotional Clarity which was, in turn, associated with lower PA; and (2) the Emotional Clarity and Emotional Repair relationships (Ind6 = *a*_2_*d*_32_*b*_3_), where greater SR was associated with lower Emotional Clarity and lower Emotional Repair which, in turn, were associated with lower PA (Figure 3). To verify which of the indirect effects is more important, we performed specific contrasts of the indirect effects (Table 4) and observed that C19 (comparing Ind5 with Ind6) was not statistically significant, β = 0022, SE = 0.0121, 95% CI [−0.0210, 0.0285]. Therefore, both indirect effects are equally important.

**Table 4:** Path coefficients, total effect, direct effect, indirect effect and main specific indirect effect contrast definitions, and 95% bias-corrected confidence interval predicting Positive affect scores (*N* = 467).

**Path**	**Coefficient**	**SE**	**BootLLCI**	**BootULCI**	***t***	***p***
Total effect (*c*)	0.2727	0.0708	0.1335	0.4119	3.85	0.000
Direct effect (*c*')	0.2911	0.0661	0.1612	0.4209	4.40	0.000
*a_1_*	0.0673	0.0567	−0.0441	0.1786	1.18	0.236
*a_2_*	−0.1766	0.0579	−0.2905	−0.0628	−3.04	0.002
*a_3_*	0.0305	0.0593	−0.0861	0.1470	0.5136	0.608
*b_1_*	0.1570	0.0540	0.0509	0.2631	2.90	0.004
*b_2_*	0.1124	0.0551	0.0041	0.2207	2.03	0.042
*b_3_*	0.3788	0.0518	0.1612	0.4209	4.40	0.000
*d*_21_	0.1420	0.0473	0.0490	0.2350	2.99	0.003
*d*_31_	−0.0319	0.0484	−0.1271	0.0633	−0.659	0.510
*d*_32_	0.3291	0.0471	0.2366	0.4215	6.99	0.000
Indirect effects	**Effect**	**SE**	**BootLLCI**	**BootULCI**		
Total indirect effect	−0.0183	0.0318	−0.0807	0.0447		
*Ind5: a_2_b_2_*	−0.0199	0.0119	−0.0508	−0.0026		
*Ind6: a_2_d_32_b_3_*	−0.0220	0.0087	−0.0432	−0.0083		
Specific indirect effect contrast definitions	**Effect**	**SE**	**BootLLCI**	**BootULCI**		
(C19) Ind5 minus Ind6	0.0022	0.0121	−0.0210	0.0285		

*BootLLCI, bootstrapping lower limit confidence interval; BootULCI, bootstrapping upper limit confidence interval; SE, standard error*.

*Model: 6*.

*Y: Positive Affect*.

*X: Sensitivity to Punishment*.

*M1: Emotional attention*.

*M2: Emotional clarity*.

*M3: Emotional repair*.

*N = 467*.

**Figure 4:**
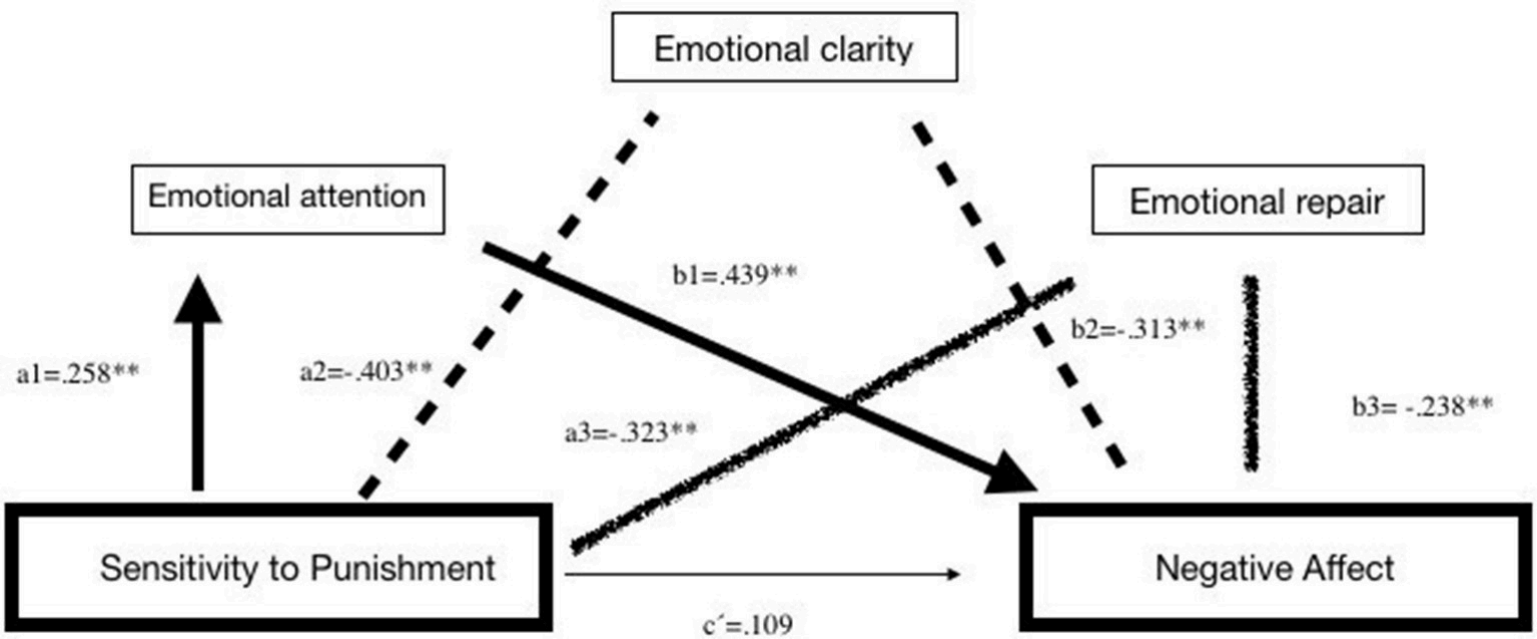
Illustration of three-way of serial mediation model between sensitivity to punishment and negative affect. **p < 0.001.

Regarding Model B, the analyses show that SP accounted for 8.83% of the unique variance of NA (*R*^2^ = 0.0883, *F* = 45.011, *p* < 0.01), but 23.45% of the total variance was accounted for by the global model (*R*^2^ = 0.2345, *F* = 35.384, *p* < 0.01). The values provided in Table 5 show that the total effect (*c*) of SC on NA was significant, and the direct effect (*c*′) of SR on PA was no significant. We obtained three specific indirect effects, all of them with the same weight: (1) through the Emotional Attention (Ind1 = *a*_1_*b*_1_), in which greater SP was associated with more Emotional Attention, which, in turn, was associated with more NA (2); through Emotional Clarity (Ind5 = *a*_2_*b*_2_); and through (3) Emotional Repair (Ind7 = *a*_3_*b*_3_), in which greater SP was associated with lower Emotional Clarity or Repair, which, in turn, were associated with greater NA (see Figure 4).

**Table 5:** Path coefficients, total effect, direct effect, indirect effect and main specific indirect effect contrast definitions, and 95% bias-corrected confidence interval predicting negative affect scores (*N* = 467).

**Path**	**Coefficient**	**SE**	**BootLLCI**	**BootULCI**	***t***	***p***
Total effect (*c*)	0.4192	0.0625	0.2964	0.5420	6.71	0.000
Direct effect (*c*′)	0.1085	0.0666	−0.0224	0.2394	1.63	0.104
*a_1_*	0.2581	0.0455	0.1687	0.3475	5.67	0.000
*a_2_*	−0.4032	0.0465	−0.4945	−0.3119	−8.68	0.000
*a_3_*	−0.3225	0.0521	−0.4249	−0.2202	−6.19	0.000
*b_1_*	0.4390	0.0603	0.3205	0.5575	7.28	0.000
*b_2_*	−0.3130	0.0606	−0.4321	−0.1939	−5.16	0.000
*b_3_*	−0.2380	0.0571	−0.3503	−0.1258	−4.17	0.000
*d*_21_	0.2351	0.0458	0.1451	0.3251	5.13	0.000
*d*_31_	0.0657	0.0489	−0.0305	0.1619	1.34	0.180
*d*_32_	0.2139	0.0483	0.1191	0.3088	4.43	0.000
Indirect effects	**Effect**	**SE**	**BootLLCI**	**BootULCI**		
Total indirect effect	0.3107	0.0454	0.2292	0.4075		
*Ind1: a_1_b_1_*	0.1133	0.0268	0.0679	0.1746		
*Ind2: a_1_b_2_*	−0.0190	0.0063	−0.0351	−0.0097		
*Ind4: a_1_d_21_d_32_b_3_*	−0.0031	0.0015	−0.0077	−0.0011		
*Ind5 = a_2_b_2_*	0.1262	0.0295	0.0743	0.1887		
*Ind6 = a_2_d_32_b_3_*	0.0205	0.0086	0.0079	0.0427		
*Ind7 = a_3_b_3_*	0.0768	0.0241	0.0351	0.1314		
Specific indirect effect contrast definitions	**Effect**	**SE**	**BootLLCI**	**BootULCI**		
(C1) Ind1 minus Ind2	0.1323	0.0301	0.0811	0.2010		
(C3) Ind1 minus Ind4	0.1164	0.0274	0.0700	0.1790		
(C5) Ind1 minus Ind6	0.0928	0.0273	0.0459	0.1552		
(C8) Ind2 minus Ind4	−0.0159	0.0060	−0.0313	−0.0070		
(C9) Ind2 minus Ind5	−0.1452	0.0336	−0.2175	−0.0856		
(C10) Ind2 minus Ind6	−0.0395	0.0103	−0.0636	−0.0229		
(C11) Ind2 minus Ind7	−0.0958	0.0246	−0.1506	−0.0531		
(C16) Ind4 minus Ind5	−0.1293	0.0293	−0.1914	−0.0778		
(C17) Ind4 minus Ind6	−0.0236	0.0099	−0.0491	−0.0091		
(C18) Ind4 minus Ind7	−0.0799	0.0248	−0.1352	−0.0367		
(C19) Ind5 minus Ind6	0.1057	0.0313	0.0502	0.1729		
(C21) Ind6 minus Ind7	−0.0562	0.0219	−0.1100	−0.0217		

*BootLLCI, bootstrapping lower limit confidence interval; BootULCI, bootstrapping upper limit confidence interval; SE, standard error*.

*Model: 6*.

*Y: Negative Affect*.

*X: Sensitivity to Punishment*.

*M1: Emotional attention*.

*M2: Emotional clarity*.

*M3: Emotional repair*.

*N = 467*.

## Discussion

In the present work, we analyzed the mediation of TEI (Attention, Clarity, and Emotional Repair) in the relationship between BIS/BAS and affective states. For this purpose, two mediation models were designed: Model A examined the effect of SR or BAS on PA, and Model B examined the effect of SP or BIS on NA. Both mediation analyses were performed using TEI as the mediator.

Our preliminary analyses suggest that BAS activity is related to the increase of PA or mood (Corr, 2004). It confirms that this system drives people to achieve their desired goals, leading to feelings of joy and positive mood when they attain them. However, the opposite occurs when people are more sensitive to BIS, which is related to a greater experience of negative mood. Due to their high negative affect and its link to certain disorders like anxiety and depression (Sandín et al., 1999), these people would be more likely to develop this type of psychopathology (Johnson et al., 2003; Leen-Feldner et al., 2004; Corr and McNaughton, 2008; Maack et al., 2012; Hundt et al., 2013).

Moreover, our results partially confirm the findings of Bacon and Corr (2017) concerning the relations between TEI and the RST, because people with low EI are more nervous and cautious in rewarding environments, that is, they have greater BIS activation. However, we did not find the same result with regard to people with high EI being more goal-directed, because we found a negative relation between SR and Emotional Clarity. We must take into account that the assessment instruments in our study are different from those used by Bacon and Corr (2017), both for measuring EI and the construct of RST.

Nevertheless, our results show that TEI is associated with a positive mood and low EI is related to a negative mood, as in other studies (Gohm and Clore, 2002; Palmer et al., 2002; Extremera and Fernández-Berrocal, 2005; Extremera and Rey, 2016).

With the idea of expanding the previous works, we focused on exploring the role of TEI as a mediator, in order to better understand the real process that takes place between the systems of personality according to the RST, TEI, and the emotional state.

In the first model, we hypothesized that greater BAS activation would be associated with higher levels of TEI, which would be related to greater PA. In this regard, our data confirm that if people are more receptive to appetitive stimulation, this leads to a more positive affective state. But its explanatory power significantly increases, rising from 3.09 to 18.17%, when people think they do not understand their emotions, or they analyze them excessively or think they are incapable of regulating or controlling them. And consequently, that positive state will decrease. In this case, we note that inadequate beliefs about one's emotional abilities, such as clarity and regulation, can change the direction of the relation between BAS and pleasant experiences of joy or positive emotions (Meyer and Hofmann, 2005; Corr, 2008a,b).

In the second model, our hypothesis was that greater BIS activation would be associated with lower levels of TEI, which, in turn, would be related to more NA. To confirm this hypothesis, we focused on the analyses of the indirect effects that emerge through emotional clarity and repair, because they reaffirm that higher activation of the inhibitory behaviors (BIS) can produce greater NA, and that this relation increases if people cannot understand or regulate their emotions. Therefore, this follows the lines proposed by various authors regarding how BIS activation is related to the behaviors of passive avoidance, contributing to the evaluation of risk and rumination, which, in turn, can lead to the experience of anxiety and which is associated with lower TEI (Corr, 2008a,b; Bacon and Corr, 2017).

However, this positive relation between SP and NA also increases if people pay excessive attention to their feelings, that is, they have a high level of emotional attention. These data would contradict our prior comments. Some studies support that high attention to emotions produces or are related to the tendency to ruminate and its possible harmful effects (Extremera and Fernández-Berrocal, 2005).

This study has some limitations, for example, those associated with the use of self-reports for data collection, besides the limitations of the cross-sectional studies and sex ratio should also be included. We recommend expanding the sample and including other study populations in order to increase the representativeness and generalizability of the data.

In future research, we propose that the studied constructs be assessed with other scales based on the RST and with other measurement instruments of EI that are supported by the skills models.

In spite of the limitations, this study makes a significant contribution to understanding the processes established between TEI, the motivational systems (BIS/BAS), and affective states. In short, the results shed light on the involvement of two motivational systems in emotional states, and how this relation is changed and better explained by TEI. Consequently, although motivators may be a focus of interest for interventions, this study shows that the addition of the TEI construct could increase the efficiency and the profitability of these practical applications.

## Author Contributions

AM-C developed the study design, survey creation, performed the collection, and the data analysis and writing of manuscript. JA-B contributed to the interpretation and writing of the manuscript and approved the final version of the manuscript for submission. AZ contributed to project design, performed the collection and manuscript revision. RG contributed to project design and data analysis, data preparation and coding and writing the manuscript, and approved the final version of the manuscript for submission.

### Conflict of Interest Statement

The authors declare that the research was conducted in the absence of any commercial or financial relationships that could be construed as a potential conflict of interest.
